# Repeatability and reproducibility of longitudinal relaxation rate in 12 small-animal MRI systems

**DOI:** 10.1016/j.mri.2019.03.008

**Published:** 2019-06

**Authors:** John C. Waterton, Catherine D.G. Hines, Paul D. Hockings, Iina Laitinen, Sabina Ziemian, Simon Campbell, Michael Gottschalk, Claudia Green, Michael Haase, Katja Hassemer, Hans-Paul Juretschke, Sascha Koehler, William Lloyd, Yanping Luo, Irma Mahmutovic Persson, James P.B. O'Connor, Lars E. Olsson, Kashmira Pindoria, Jurgen E. Schneider, Steven Sourbron, Denise Steinmann, Klaus Strobel, Sirisha Tadimalla, Irvin Teh, Andor Veltien, Xiaomeng Zhang, Gunnar Schütz

**Affiliations:** a Bioxydyn Ltd, Manchester Science Park, Rutherford House, Pencroft Way, MANCHESTER M15 6SZ, United Kingdom; b Centre for Imaging Sciences, Division of Informatics Imaging & Data Sciences, School of Health Sciences, Faculty of Biology Medicine & Health, University of Manchester, Manchester Academic Health Sciences Centre, MANCHESTER M13 9PL, United Kingdom; c Merck & Co., Inc., West Point, PA, United States of America; d Antaros Medical, BioVenture Hub, 43183 Mölndal, Sweden; e MedTech West, Chalmers University of Technology, Gothenburg, Sweden; f Sanofi-Aventis Deutschland GmbH, R&D TIM - Bioimaging Germany, Industriepark Höchst, D-65926 Frankfurt am Main, Germany; g Bayer AG, Research and Development, Pharmaceuticals, MR and CT Contrast Media Research, Müllerstraße 178, D-13353 Berlin, Germany; h In-Vivo Bioimaging UK, RD Platform Technology & Science, GSK Medicines Research Centre, Gunnels Wood Road, STEVENAGE, Hertfordshire, SG1 2NY, United Kingdom; i Lund University BioImaging Center, Klinikgatan 32, SE-222-42 Lund, Sweden; j Bruker BioSpin MRI GmbH, Rudolf-Plank-Straße 23, D-76275 Ettlingen, Germany; k iSAT Discovery, Abbvie, 1 North Waukegan Road, North Chicago, IL, 60064-1802, United States of America; l Department of Translational Sciences, Medical Radiation Physics, Lund University, Skåne University Hospital, SE-205 02 Malmö, Sweden; m Division of Cancer Sciences, School of Medical Sciences, Faculty of Biology Medicine & Health, University of Manchester, Manchester Academic Health Sciences Centre, MANCHESTER M20 4BX, United Kingdom; n Leeds Institute of Cardiovascular and Metabolic Medicine, University of Leeds, Leeds LS2 9JT, United Kingdom; o Leeds Imaging Biomarkers Group, Department of Biomedical Imaging Sciences, University of Leeds, LIGHT Labs, Clarendon Way, LEEDS LS2 9JT, United Kingdom; p Radboud university medical center, Radiology (766), P.O.Box 9101, 6500, HB, Nijmegen, the Netherlands

**Keywords:** Av, Avance, B_0_, applied magnetic field, CoV, coefficient of variation, DNE, dynamic no enhancement, FISP, fast steady-state free-precession, ICH GCP, International Conference on Harmonisation of Technical Requirements for Registration of Pharmaceuticals for Human Use Harmonised Tripartite Guideline for Good Clinical Practice, PV, ParaVision, *R*_1_, longitudinal relaxation rate, *r*_1_, longitudinal relaxativity, rms, root-mean-square, SNR, signal-to-noise ratio, *T*_1_, longitudinal relaxation time, T1W, *T*_1_-weighted, *T*_2_, transverse relaxation time, MRI, Hardware stability, Biomarker, Relaxation time, Phantom, Reproducibility, Error propagation

## Abstract

**Background:**

Many translational MR biomarkers derive from measurements of the water proton longitudinal relaxation rate *R*_1_, but evidence for between-site reproducibility of *R*_1_ in small-animal MRI is lacking.

**Objective:**

To assess *R*_1_ repeatability and multi-site reproducibility in phantoms for preclinical MRI.

**Methods:**

*R*_1_ was measured by saturation recovery in 2% agarose phantoms with five nickel chloride concentrations in 12 magnets at 5 field strengths in 11 centres on two different occasions within 1–13 days. *R*_1_ was analysed in three different regions of interest, giving 360 measurements in total. Root-mean-square repeatability and reproducibility coefficients of variation (CoV) were calculated. Propagation of reproducibility errors into 21 translational MR measurements and biomarkers was estimated. Relaxivities were calculated. Dynamic signal stability was also measured.

**Results:**

CoV for day-to-day repeatability (*N* = 180 regions of interest) was 2.34% and for between-centre reproducibility (*N* = 9 centres) was 1.43%. Mostly, these do not propagate to biologically significant between-centre error, although a few *R*_1_-based MR biomarkers were found to be quite sensitive even to such small errors in *R*_1_, notably in myocardial fibrosis, in white matter, and in oxygen-enhanced MRI. The relaxivity of aqueous Ni^2+^ in 2% agarose varied between 0.66 s^−1^ mM^−1^ at 3 T and 0.94 s^−1^ mM^−1^ at 11.7T.

**Interpretation:**

While several factors affect the reproducibility of *R*_1_-based MR biomarkers measured preclinically, between-centre propagation of errors arising from intrinsic equipment irreproducibility should in most cases be small. However, in a few specific cases exceptional efforts might be required to ensure *R*_1_-reproducibility.

## 1 Introduction

Many useful MR biomarkers derive from measurements of the water proton longitudinal relaxation time *T*_1_, or alternatively the relaxation rate *R*_1_ ≡ *T*_1_^−1^. Errors in *R*_1_ [1] are common, will propagate, and may damage the reproducibility and accuracy of the resulting MR biomarkers. Although considerable effort has been devoted to measuring and assuring the accuracy of *R*_1_ in clinical MR [[2], [3], [4], [5]] systems, there is little evidence for the cross-site reproducibility of *R*_1_ measurements in MR systems designed for small-animal research. The lack of standardisation in preclinical imaging has been recognised as an important problem [6,7] which in the worst case could invalidate the findings from animal studies, or confound meta-analyses and translation.

Reproducibility in a valid phantom is an important and ethical prerequisite for reproducible values *in vivo*. Poor technical validation has been a major impediment to clinical translation of MR biomarkers [8]. An ideal *R*_1_ phantom should be traceable [2]; resist biological, chemical and physical deterioration; perform effectively over a range of temperatures convenient and relevant for the users; cover the parameter range expected in subsequent studies; not exhibit physiologically unrepresentative MR characteristics such as radiation damping, convection, unphysiologic *T*_2_, excessive self-diffusion, off-resonance chemical shifts, standing waves, or abrupt boundaries; interrogate the entire volume subsequently to be occupied by body parts being imaged; have dimensions suitable for the subject subsequently to be imaged (in this case rats and mice); be convenient for the intended users; and be cost-effective for the intended users. To meet these criteria, nickel agarose phantoms following the design of Christoffersson et al. [9] were used.

Two distinct general approaches to MR standardisation have previously been employed. In the first, which we term “centrally-led”, a central organisation, often independent of the participating sites, is accountable for overall measurement accuracy and reproducibility. They mandate the phantom and acquisition protocol and analyse centrally. They may perform set-up and training at each participating site, instruct sites to repeat aberrant measurements, or even expel sites who cannot achieve the required accuracy. Centrally-led standardisation is common in clinical trials performed to ICH GCP [10,11], or where the MR measurement is regulated as a companion diagnostic [12]. In the second approach, which we term “institution-led”, each investigator is accountable for measurement accuracy in their own centre. They are responsible for their own acquisition and analysis, and for compliance with any guidelines for their chosen phantom. “Institution-led” standardisation is common in academic research and in single-centre studies. Although we expect “centrally-led” standardisation to provide better reproducibility than “institution-led” standardisation, in this work we modelled “institution-led” standardisation as this is more representative of practice in preclinical MR. The study was performed within an international consortium of imaging centres participating in the validation of imaging biomarkers [13], and developing reliable preclinical MR assays which would give comparable results in different laboratories. The aim of this work was to assess the repeatability and reproducibility of *R*_1_ in a realistic rodent MR protocol. Simple simulations were performed in order to compare the likely propagation of reproducibility errors into a broad range of *R*_1_-derived MR biomarkers.

## 2 Materials and methods

### 2.1 Preclinical phantom

Batches of 2% agarose with nickel chloride concentrations respectively of 0.50, 1.04, 2.02, 4.08 and 8.05 mM, with 0.05% sodium azide, were prepared centrally in Berlin and used to create identical phantoms (Supplementary Fig. S3.1) which were distributed to the participating laboratories. The phantoms were prepared and authenticated (supplementary material S3) in July 2017, shipped in August 2017, and the measurements were performed between December 2017 and February 2018.

### 2.2 MR methods

Thirteen centres involved in an international consortium for the validation of imaging biomarkers for drug safety assessment [13] were invited to participate. Where centres had access to more than one MR system, they were invited to submit data from multiple MR systems. Eleven centres agreed to participate, one of which (G) provided data from two different magnets (G1 and G2): in the analyses, G1 and G2 were treated as if from two different centres. Details of the 12 MR systems are given in Table 1. Eleven of the 12 MR systems (all except B) were in laboratories which regularly and routinely measure MR biomarkers in rodents, intending to translate their findings to create diagnostics or therapeutics to improve human health. Although the use of any particular manufacturer's equipment was not mandated, all participating centres elected to employ Bruker Avance/ParaVision systems. An “institution-led” approach to standardisation was adopted. Pilot studies were performed only in centres B and G. No site training was performed, no quality control was imposed, nor were sites permitted to repeat their measurements to eliminate apparent outliers. Region-of-Interest (RoI) definition and *T*_1_ calculation were performed locally.

**Table 1:** Equipment used. All equipment was manufactured by Bruker (Rheinstetten, Germany) using Avance (Av) spectrometers and ParaVision (PV) acquisition and analysis software except: (a) Magnet from the companies which formerly traded as Varian, Magnex or Agilent; (b) Transmitter-Receiver from Rapid MR International, Columbus OH USA or Rimpar, Germany.

Centre	B_0_/T	Spectrometer	Gradient strength/mT∙m^−1^ (model)	Radiofrequency transmitter/receiver volume coil (i.d./mm)	Software
A	7^a^	Pharmascan 70/16 US Av III	375 (B-GA9S)	Quadrature 300 MHz (38)^b^	PV 6.0
B	3	Biospec 3 T Av IIIHD	900 (B-GA105S HP)	Quadrature 128 MHz (60)	PV 6.0.1
C	7	Biospec 70/20 USR Av IIIHD	660 (B-GA12S HP)	Quadrature 300 MHz (86)	PV 6.0.1
D	4.7	Biospec 47/20 USR Av IIIHD	660 (B-GA12S HP)	Quadrature 200 MHz (72)	PV 6.0.1
E	7	Biospec 70/30 USR Av II	440 (B-GA12S)	Single channel 300 MHz (72)	PV 6.0.1
F	7^a^	Biospec 70/20 Av I	400 (B-GA12)	Single channel 300 MHz (72)	PV 5.1
G1	7	Biospec 70/30 USR Av III	300 (B-GA12)	Quadrature 300 MHz (90)^b^	PV 6.0.1
G2	4.7	Biospec 47/40 Av III	200 (B-GA12S)	Quadrature 200 MHz (90)^b^	PV 6.0.1
H	4.7	Pharmascan 47/16 Av III	300 (B-G9S)	Single channel 200 MHz (60)	PV 5.1
J	4.7	Biospec 47/40 USR Av II	660 (B-GA12S HP)	Quadrature 200 MHz (72)	PV 6.0.1
K	9.4^a^	Biospec 94/30 Av III	670 (B-GA 12S HP)	Quadrature 400 MHz (87)	PV 6.0.1
L	11.7	Biospec 117/16 USR Av III	750 (B-GA 9S)	Quadrature 500 MHz (72)	PV 6.0.1

Centres were asked to measure *R*_1_ by saturation recovery using a standard RARE sequence. (Additional measurements using an investigational fast steady-state free-precession (FISP) sequence designed for the consortium's *in vivo* needs will be reported elsewhere). In an attempt to provide temperature stability and minimise susceptibility artefacts, each phantom was embedded in a cucumber (Supplementary Figs. S3.2 and S3.3). Centres were instructed to “allow the five cucumbered phantoms to come to thermal equilibrium in the magnet bore…[and] measure the temperature of the cucumber flesh in several places and verify thermal equilibrium has been reached.” The temperature of the cucumber flesh around the phantom was measured before and after each acquisition. The entire protocol was run in each centre on two separate days, mean 2.7 days apart (range 1–13).

In ParaVision, the standard RARE *T*_1_ saturation-recovery measurement method “T1map_RARE protocol” (Rat/Head/Relaxometry) was invoked. All images were coronal with 58 × 58 mm field of view, 128 × 128 matrix, with a π/2 for 1.16 mm slice selection followed by a π train with RARE factor 8, effective echo time 30 ms, echo spacing 7.5 ms. Signal averaging was not employed and 5 dummy scans were used. Saturation recovery experiments used repetition times TR of 5500, 2000, 1200, 750, 500, 300, 200 and 100 ms giving a scan time of 169 s, not including the dummy scans. Next, a “dynamic-no-enhancement” (DNE) stability series to simulate dynamic contrast-enhanced MRI was run for 5 min (approximately 34 images) with repeated acquisition using the same parameters but with TR fixed at 500 ms.

### 2.3 Analyses

Each centre conducted measurements independently and was blinded to findings from the other centres until their own results had been submitted. At each centre, *T*_1_ values were obtained using a 2-parameter fit in ParaVision from circular 25 mm^2^ RoIs, *i.e.* 29 μl volumes, approximately 120 voxels, at three RoI positions. These were: at the isocentre; radially at the edge of the phantom 10 mm from isocentre; and axially at the end of the phantom 12–20 mm from isocentre, denoted respectively by (X,Y,Z) = (0,0,0), (10,0,0) and (0,0,12) mm. The 2-parameter fit assumed zero longitudinal magnetisation at the mid-point of the eighth echo. The resulting *T*_1_ values and standard deviation of the fit for each RoI, together with the mean and standard deviation DNE signal for (X,Y,Z) = (0,0,0), were submitted to the core lab in Manchester for further analysis.

At the core lab, root-mean-square (rms) within-centre *R*_1_ repeatabilities and between-centre reproducibilities were calculated using Microsoft Excel. Each calculation was performed both using absolute units (*i.e.* standard deviations with units s^−1^), and using coefficients of variation (CoV, dimensionless, presented as percentages). This was done because absolute *R*_1_ units (s^−1^) propagate to absolute concentration of relaxive substance and in some instances to absolute biomarker value, while coefficients of variation may be more relevant when biomarker change is considered. Post-hoc tests of significance were made for “effect of day” using Student's *t*-test, and for “effect of RoI position” by analysis of variance. No correction for multiple comparisons was made but p < 0.01 was considered significant. For each centre, weighted mean *R*_1_ values were calculated for each of the five phantoms:

$$R_{1}=\left(\sum_{d=1,2}\;\sum_{\mathrm{RoI}=0,X,Z}\left(w_{d,\mathrm{RoI}}\times R_{1,d,\mathrm{RoI}}\right)\right)/\left(\sum_{d=1,2}\;\sum_{\mathrm{RoI}=0,X,Z}w_{d,\mathrm{RoI}}\right)$$

where *R*_1,*d,RoI*_ are the *R*_1_ values for each of the two days in each of the three RoIs, and *w*_*d,RoI*_ are the corresponding weights, derived from the *T*_1_ fit in ParaVision:

$$w={\left(\text{fitted slope}/\mathrm{SD}\;\text{of}\;\mathrm{fit}\right)}^{2}$$

These weighted mean *R*_1_ values were then used to obtain relaxivities by linear regression:

(1) $$R_{1}/s^{-1}=r_{1,B_{0}}\times \left[\mathrm{Ni}\right]+R_{1,\left[\mathrm{Ni}\right]=0,B_{0}}+\varepsilon$$

where *r*_1, B_0__/s^−1^ ∙ mM^−1^ is the longitudinal relaxivity of aqueous Ni^2+^ in 2% agarose at field B_0_, *R*_1, [Ni]=0, B_0__/s^−1^ is the longitudinal relaxation rate of 2% agarose at field B_0_, and *ε* is a normally-distributed error term assumed to subsume *inter alia* any temperature effects.

### 2.4 Cross-validation

Our “institution-led” study design required each centre to derive its own *T*_1_ values. Since centres elected to use the proprietary ParaVision software, a small supplementary study was also performed using an alternative analysis to verify values. Data from one centre were reanalysed. Centre A's data were considered a good test set because they submitted data with both high and low fit errors. For each of the 10 RARE data sets (5 phantoms × 2 days), and for the same three RoIs used in the primary analysis, signal mean and standard deviation were retrieved for each TR value. *R*_1_ was calculated using “R” [14] using four expressions of the form:

  $$\text{model}<-\mathrm{nls}\left(y\left[,i\right]\sim I\left(\text{Minf}-\left(\text{Minf}-M0\right)*\exp\left(-R1^{*}\left(\mathrm{TR}-0.06\right)\right)\right),\text{weights}=\left(w\left[,i\right]\right)\ldots \right)$$

For three-parameter fits, Minf, M0 and R1 were fitted, while for two-parameter fits M0 was set to zero. For weighted fits, each RoI value y was weighted by w, the inverse of the variance in y, while for unweighted fits w was set to unity. For each of the 30 data sets, each of the four estimates of *R*_1_ from “R”, *R*_1_^R^, was compared with the reciprocal *T*_1_ from Paravision, *R*_1_^PV^. In each of the four cases:

(2) $$\text{mean difference}=\frac{1}{30}\sum \frac{R_{1}^{\mathrm{PV}}-R_{1}^{R}}{\left(R_{1}^{R}+R_{1}^{\mathrm{PV}}\right)/2}\times 100\%$$

### 2.5 Illustrative simulations

Error propagation associated with two standard deviations of *R*_1_ reproducibility was estimated for a range of derived measurements and biomarkers, using representative relaxivities and other parameters from the literature. This is conservative as it does not fully eliminate repeatability error. Three general cases were considered: firstly, native *R*_1_ (or *T*_1_) used as a biomarker; secondly, concentration of endogenous or exogenous paramagnetic substance used as biomarker; and thirdly, biomarkers derived from compartmental models. For Dynamic Contrast-Enhanced (DCE) MRI, the error in precontrast *R*_1_ was propagated into the biomarkers for four preclinical case-studies. Representative ‘true’ values of kinetic parameters, pre-contrast *R*_1_ values, and appropriate tracer kinetic models were chosen from literature to estimate contrast agent concentration uptake in each tissue type. Simulation parameters are provided in Supplementary Material.

## 3 Results

**Fig. 1:**
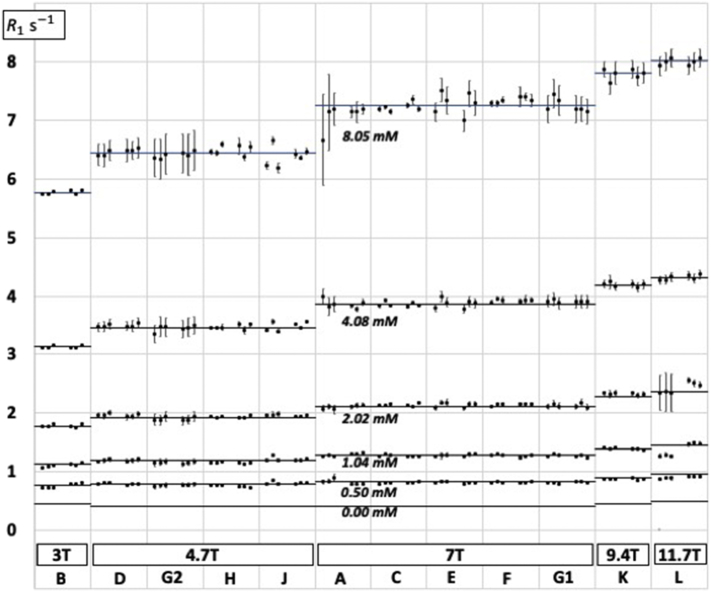
*R*_1_ measurements (logarithmic axis) for each of centres A–L. Each centre made measurements on five 2% agarose phantoms with different Ni^2+^ concentrations. The six horizontal lines represent *R*_1_ values calculated from the field-dependent relaxivities as explained in Table 2. There are two groups of three data points for each phantom at each centre representing, respectively, days 1 and 2, and RoIs (X,Y,Z) = (0,0,0), (10,0,0) and (0,0,12). Error bars are *T*_1_ fit errors from ParaVision.

Each centre was requested to submit 30 *R*_1_ measurements (5 phantoms × 3 locations × 2 days), the results of 10 DNE runs (5 phantoms × 2 days), and the 10 associated temperature measurements (5 phantoms × 2 days). The quality of the exponential fits for the 8 TR values was generally good, although in 15/360 cases the fit error was worse than 5% (9 cases in centre G2, 3 cases in centre A and 3 cases in centre L) (see Fig. 1). All these outliers were included in the analysis and not eliminated. One centre (J) did not provide DNE or temperature measurements in a suitable format, so its results were omitted from any analyses that needed those data. For the other centres, temperatures were recorded to ±0 .1 °C: the mean was 19.3 °C (SD 1.3), the mean deviation in temperature between day 1 and day 2 was 0.65 °C, and the worst deviation 5 °C (centre B, 0.5 mM phantom).

### 3.1 Longitudinal relaxation rates and relaxivities

**Fig. 2:**
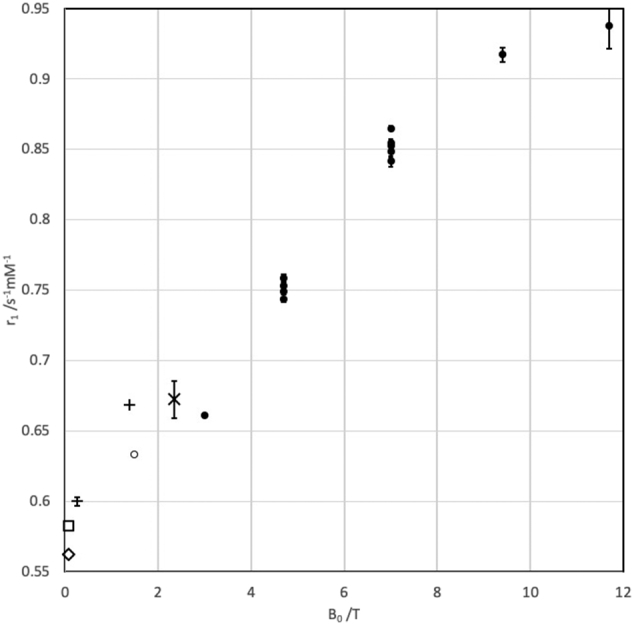
Plot of [Ni^2+^] relaxivities in 2% agarose against field strength. Closed circles: this work, 19.36 ± 1.20 °C. Open circle: data from initial 1 5 T characterization of the phantom materials (see supplementary material), 21.5 °C. Standard error of fit is shown, although for B_0_ between 1 5 T and 7 T the standard errors of between 0.19% and 0.48% are not evident as they are smaller than the size of the symbol. Other symbols: estimated from literature. **+**, parameter *c*_1_ in [3], 22 °C. −, estimated, with standard error, from Fig. 1 in [9], 22 °C. **×**, estimated, with standard error, from Fig. 4 in [15], 20 °C. ◇, ◻︎, estimated from Fig. 2 in [16], 19 °C and 22 °C respectively.

Fig. 1 depicts the individual *R*_1_ data, and Table 2 provides mean values. Fig. 2 shows the field dependence of *r*_1_ from this work, with additional data points added from the literature [3,9,15,16].

**Table 2:** Relaxation rates *R*_1_ and relaxivities *r*_1_. At each centre *R*_1_ (measured) represents the weighted mean of the six measurements (2 days × 3 positions), while *R*_1_ (fitted, 0.00 mM) and *r*_1_ are respectively the intercept and slope of a linear regression of *R*_1_ against [Ni^2+^]. At 4.7 T and 7 T, where measurements were made at multiple centres, the SD is also given.

	3.0 T	4.7 T (SD) N = 4	7.0 T (SD) N = 5	9.4 T	11.7 T
*R*_1_ (measured)/s^−1^					
0.50 mM	0.768	0.779 (0.023)	0.808 (0.012)	0.866	0.898
1.04 mM	1.123	1.171 (0.023)	1.276 (0.012)	1.385	1.386
2.02 mM	1.782	1.934 (0.026)	2.131 (0.013)	2.330	2.518
4.08 mM	3.126	3.474 (0.019)	3.881 (0.037)	4.189	4.313
8.05 mM	5.762	6.443 (0.038)	7.248 (0.065)	7.808	8.002
*R*_1_ (fitted)/s^−1^					
0.00 mM	0.438	0.404 (0.027)	0.394 (0.006)	0.438	0.481
Relaxivity *r*_1_/s^−1^ mM^−1^	0.661	0.751 (0.006)	0.852 (0.009)	0.917	0.938

### 3.2 Repeatability, reproducibility and linearity

Table 3 shows repeatability and reproducibility. Day-to-day repeatability ranged from 0.025 s^−1^ (centre D) to 0.097 s^−1^ (centre A): day-to-day repeatability CoV ranged from 0.76% (centre F) to 5.48% (centre L). In exploratory analysis, the day-to-day repeatability of 2.34% was not markedly improved either if measurements were restricted to the isocentre (2.22%), or if measurements with >1 °C difference in temperature between day 1 and day 2 were excluded (2.05%). No evidence was seen for field dependence of repeatability. For day-to-day repeatability, 2 centres (B, L) showed a statistically significant effect of day, and 4 centres (D, E, G2, K) showed a statistically significant effect of RoI position. Dynamic (DNE) signal stability CoV varied between 0.30% (centre C) and 2.1% (centre L), and in exploratory analyses was not found to be associated with B_0_, nor with repeatability, nor with the *T*_1_ fit error. Between-centre reproducibility of *R*_1_ was measured for the 5 phantoms at both 4.7 T and 7 T. Least reproducible, on a CoV basis, was the 0.5 mM phantom at 4.7 T (2.94%, *N* = 4 centres) or, on an absolute units basis, the 8 mM phantom at 7 T (0.065 s^−1^, *N* = 5 centres). In exploratory analysis, reproducibility was not improved if measurements were restricted to the isocentre (all-RoIs rms reproducibility was 0.031 s^−1^ or 1.4% while isocentre rms reproducibility was 0.064 s^−1^ or 1.6%).

**Table 3:** Repeatability and reproducibility. CoV: coefficient of variation; rms: root mean square. The DNE row shows signal stability for a “dynamic-no-enhancement” (DNE) run of *T*_1_-weighted (T1W) acquisitions.

	Number of centres	Number of measurements aggregated	rms error	
			Absolute	CoV
Repeatability				
*R*_1_ fit error	12	360	0.105 s^−1^	1.87%
*R*_1_ day-to-day	12	180 × 2	0.056 s^−1^	2.34%
*R*_1_ isocentre *vs.* off-centre	12	120 × 3	0.059 s^−1^	2.22%
DNE T1W signal	11	110 × 34	–	0.84%
Reproducibility				
*R*_1_ centre-centre	9	45	0.031 s^−1^	1.43%
*R*_1_ centre-centre (isocentre only)	9	45	0.064 s^−1^	1.56%
Relaxivity centre-centre	9	9	0.008 s^−1^mM^−1^	0.83%

A measure of the linearity of *R*_1_ as a biomarker over the range 0.8–8 s^−1^ was obtained from the relaxivity Eq. (1): the rms standard error of *r*_1,B_0__ was 0.6% (range 0.2% in centre B, to 1.7% in centre L, *N* = 12 centres).

### 3.3 Comparison of analysis algorithms

*R*_1_ values for centre A derived from two-parameter fits performed in “R” and in Paravision were close: mean differences were 0.024% for an unweighted fit and 0.26% for a weighted fit. When three-parameter fits performed in “R” were compared with two-parameter fits performed Paravision, disagreement was greater: 1.67% for an unweighted fit and 1.74% for a weighted fit. Bland-Altman style plots are provided in Supplementary Fig. S5.

### 3.4 Illustrative propagation to irreproducibility in biomarker values

Illustrative between-centre irreproducibility expected from two standard deviations of the observed *R*_1_ reproducibility for a range of derived measurements and biomarkers are given in Table 4.

**Table 4:** Propagation of errors using Table 3 reproducibility, with plausible or representative values for a range of important measurements and biomarkers. Actual error propagation varies widely between applications: the values here should therefore be regarded as indicative, but not as a substitute for a thorough analysis of error propagation in any particular setting.

Measurement or biomarker	Reproducibility error propagated from 2SD of *R*_*1*_	Notes
Native *R*_1_ (or *T*_1_)	0.062s^-1^	
Tissue temperature	1.6–4.6°C	a
Contrast agents		
Small non-protein-bound agents e.g. gadoterate, gadopentetate, gadobutrol, relaxivities 3–11s^-1^mM^-1^ [[62], [63], [64], [65], [66], [67]].	6–21μM	
Gadobutrol in plasma at 9.4T [65], relaxivity 4.7s^-1^mM^-1^	13μM	
Gadoxetate, relaxivity [68] 5–17s^-1^mM^-1^	4–12μM	
Ferumoxytol iron oxide nanoparticles, relaxivity [69] of 20s^-1^(mM Fe)^-1^ at 1.5 T, monodisperse particle weight of 750kDa [70].	3μM (Fe) or 0.2nM (particles)	b
Investigational folate dendrimer contrast agent with relaxivity [57] 1646s^-1^mM^-1^	38nM	c
Other substances		
Deoxyhaemoglobin monomer, relaxivity [56] 0.008s^-1^mM^-1^	7.8mM	d
Tempol (investigational radioprotectant), relaxivity [71] 0.2s^-1^mM^-1^	0.3mM	e
Dissolved dioxygen, relaxivity [72] 0.1-0.3s^-1^mM^-1^	160–470mmHg	f
Derived biomarkers		
Transfer constant *K*^*trans*^ for gadopentetate in rodent glioma, extended Tofts model [[73], [74], [75]]	0.004min^-1^ (8%)	g, h
Extracellular extravascular fraction *v*_*e*_ in rodent glioma, extended Tofts model [[73], [74], [75]]	0.024 (10%)	g
Plasma fraction *v*_*p*_ in rodent glioma, extended Tofts model [[73], [74], [75]]	0.0016 (10%)	g
Transfer constant *K*_*i*_ for gadopentetate in transient ischaemia model, Patlak analysis [73,76,77]	0.0002ml.g^-1^s^-1^ (5%)	g
Plasma fraction *v*_*p*_, transient ischaemia model, Patlak analysis [73,76,77]	0.0008 (5%)	g
Flow F_p_, normal rodent lung, model-free deconvolution [73,78,79]	0.03min^-1^ (8%)	g
Plasma fraction *v*_*p*_, normal rodent lung, model-free deconvolution [73,78,79]	0.04 (10%)	g
Normal hepatocyte transporter uptake rate constant *k*_1_ for gadoxetate, 2-compartment liver model [73,[80], [81], [82]]	0.0013mM.s^-1^ (4%)	g, i
Normal hepatocyte transporter efflux rate constant *k*_2_ for gadoxetate, 2-compartment liver model [73,[80], [81], [82]]	0.0001s^-1^ (2%)	g
Extracellular extravascular fraction *v*_*e*_, 2-compartment liver model [73,[80], [81], [82]]	0.016 (7%)	g
Albumin concentration	24μM (~5%)	j
Extracellular matrix Fixed Charge Density	8mM (~4%)	k

Notes:

a Published data [[53], [54], [55]] suggest temperature dependence of tissue *R*_1_ in the range 0.013–0.0 39 s^−1^/°C.

b Note that this figure reflects longitudinal relaxivity: transverse relaxivity for this agent is higher so may provide better sensitivity. The particle molarity is only correct if monodispersity is assumed.

c This very high relaxivity is per dendrimer molecule, not per Gd.

d The physiologic range is up to 17.5 g∙dL^−1^ (11 mM).

e Tempol has been given topically at 400 mM to humans [83] and i.p. at 1.45 mmol/kg to mice [84]. Blood levels reached 3 μM in humans and 3.5 mM in mice.

f The physiologic range is 0–100 mmHg in normoxia, 0–600 mmHg in hyperoxia, >1000 mmHg with hyperbaric oxygen.

g See supplementary material

h Typically drops in *K*^*trans*^ of >20% are pharmacologically significant [59]

i A drop in *k*_1_ of 78%–96% was toxicologically significant [80]

j For an albumin concentration of around 500 μM, based on Eq. (13) and parameters from Fig. 1 in [85]. The physiologic and pathophysiologic range is approximately 450–750 μM.

k Using Eq. (3) and cartilage data from Fig. 2 in [86] These authors state “…assuming a 10% decrease in *T*_1_ is measurable…we would expect to be sensitive to a change in FCD from a normal of −0.2 to −0.16 M, the sort of change one would expect to see relatively early in a degenerative process”.

For measurements of concentration of substance, the propagated irreproducibility naturally varies with relaxivity, while for “derived” biomarkers the propagated irreproducibilities were generally ≤10%.

## 4 Discussion

In this work we addressed the repeatability and reproducibility of *R*_1_ in MR systems designed and employed for translational *in vivo* research. We prefer to work with *R*_1_ rather than *T*_1_, since from a metrology perspective [17], *R*_1_ is a ratio variable while *T*_1_ is merely an interval variable. No single method for measuring *R*_1_ is optimal for all *in vivo* studies. The most accurate methods (*e.g.* inversion recovery with long TR and short TE readout) are neither fast nor efficient. *In vivo* studies involve complex tradeoffs between accuracy, speed, spatial resolution, field of view, need for fat suppression, sensitivity to inflow, sensitivity to motion artefact, biexponential decay, and other confounding behaviours of tissue magnetisation such as *T*_2_ and magnetisation transfer. Moreover, even after a specific method is chosen, errors can be very sensitive to pulse sequence parameters such as choice of delays and nutation angles, spoiling and refocussing strategies, mis-set pulses and so on. In this study we elected to use a RARE saturation recovery technique covering the entire field of view, as this is fairly robust and efficient: our findings may not be directly translatable to other commonly used techniques such as Variable Flip Angle [1,18,19] or Look-Locker [1,20,21] which are vulnerable to different confounds, or even to other saturation-recovery techniques with different pulse sequence parameters.

### 4.1 Repeatability and reproducibility

Previous work in preclinical MR systems has addressed the between-centre reproducibility of apparent diffusion coefficients [22] and volumetrics [23], but there is little evidence on relaxation rates. Clinical MR systems are designed, maintained and operated under Medical Device regulations, but these engineering and regulatory constraints do not apply to preclinical systems, so their reproducibility might differ from clinical reproducibility.

Repeatability [24,25] (ISO 3534:2:3.3.5) refers to the similarity between measurements over a short interval made using the same test object in the same equipment operated by the same investigator. Repeatability is particularly important when the same MR biomarker is measured on successive occasions in the same human or animal, for example before and after treatment. Repeatability depends on signal-to-noise ratio and on factors such as motion artefact, for which phantoms do not model *in vivo* studies. Reproducibility [24,25] (ISO 3534:2:3.3.10) refers to the similarity between measurements made using test objects in different equipment operated by different investigators. Reproducibility is particularly important when an MR biomarker is measured once in each individual, for example in making a treatment decision in personalised healthcare. The ultimate motivation of this project is to use MR biomarkers to indicate a harmful effect of a drug, in settings where pre-treatment measurements might be unavailable, so reproducibility is the important metric. More generally, it is important to demonstrate reproducibility for multiple animal studies in different laboratories [26] to address the perceived “reproducibility crisis” [27] in translational medicine [28]. In this work, relevant values of *R*_1_ reproducibility and repeatability were small, and there was no obvious factor (such as temperature, B_0_, *R*_1_, or centre) that made any one set of measurements worse. Indeed, the error in the exponential fit of signal intensity against TR was numerically the largest error. Several between-centre studies of *T*_1_ or *R*_1_ reproducibility have been published for clinical equipment [4,5,29,30]: our CoV of 1.43% compares favourably with CoVs recently reported for inversion recovery phantom protocols in clinical systems of 5.5%–8.2% [29].

The relaxivity of Ni(H_2_O)_6_^2+^ arises because two of the 3d nickel orbitals are half-filled, creating a high-spin triplet state with two unpaired electrons. At lower fields, below 1 T, populations of the three electron spin states are almost independent of B_0_, as the Zeeman splittings are dominated by spin-orbit coupling (zero field splitting) and not by the applied field B_0_. Above 2 T, the Zeeman splittings increase linearly with B_0_. The relaxivity occurs through proton-electron dipolar mechanisms, with the relevant spectral density being the longitudinal relaxation rate *R*_1,e_ of the nickel electrons [31]. At low B_0_, *R*_1,e_ depends on fluctuations of the zero field splitting which are independent of B_0_, and previous investigators, working at relatively low fields, reported little field dependence for nickel agarose water proton *T*_1_ values [15]. However our data, taken together with previous work (Fig. 2), clearly show a modest increase in relaxivity over the range 0.1–11.7 T.

### 4.2 Implications for translational research

Repeatability errors (same subject, same device) have previously been extensively studied. Good repeatability in phantoms is a necessary, but not sufficient, condition for good repeatability *in vivo*, because phantoms seldom model physiologic variability. However reproducibility errors (between centres) are much less studied, but are critically important in translating from single-centre to multi-centre use. Since physiologic variability is largely absorbed in the repeatability error, phantoms can be very informative about reproducibility.

Water proton *T*_1_ was arguably the first MR biomarker [[32], [33], [34], [35], [36]]. Native *T*_1_ has been reported as a biomarker *inter alia* in cardiac diseases [37,38], liver diseases [35,39,40], neurology [41], oncology [34,42], in the placenta [43] and in the lung [[44], [45], [46]]. Clinically significant *R*_1_ differences (Table 4) usually exceed the expected irreproducibility reported in Table 3. For example: in liver fibrosis 0.1–0.2 s^−1^ or 10–20% [35,39,40]; in manganese neurotoxicology 0.06 s^−1^ or 7% [47]; in chronic obstructive pulmonary disease 0.1 s^−1^ or 10% [44] were clinically significant. In preclinical tumour models, differences of 15–20% were biologically significant [34]. Notably, however, in myocardial fibrosis, differences as small as 0.02–0.03 s^−1^ or 2–3% may be clinically significant [48,49] and in multiple sclerosis normal-appearing white matter differences of 0.025 s^−1^ or 2% may be clinically significant [50], so translational animal studies of these conditions may require exceptional efforts to ensure *T*_1_ measurements can be validated and qualified for decision-making for these specific indications.

A second class of imaging biomarkers attaches a specific interpretation of the observed longitudinal relaxation, for example in arterial spin labelling [51,52] or in MR thermometry [[53], [54], [55]]. Thirdly, *R*_1_ is commonly used to determine the spatially resolved *in vivo* concentration of an exogenous or endogenous paramagnetic substance of known relaxivity. Relaxivity can be field-, tissue- and temperature- dependent, and varies over many orders of magnitude between relaxive substances: from <10^−2^ s^−1^ mM^−1^ for deoxyhaemoglobin monomer [56] to >10^3^ s^−1^ mM^−1^ reported for certain investigational polymetallated contrast agents [57]. *R*_1_ errors propagate to low micromolar errors in typical gadolinium- or manganese-based contrast agents. However, propagation of errors may be more significant for techniques based on lower-relaxivity substances. For example in oxygen-enhanced MRI, which measures hyperoxia-induced changes in deoxyhaemoglobin and dissolved oxygen concentration *via* change in *R*_1_ [45,58], meticulous standardisation is warranted. From Table 4, error propagation might also be important for studies of therapeutic nitroxyls and perhaps for thermometry.

Finally, there are many biomarkers derived indirectly from contrast agent concentration, using a physiologic model. These include measures of perfusion and permeability in tumours, infarcts, synovitis or lung disease; myocardial extracellular volume, cartilage fixed charge density in osteoarthritis, and liver transporter function in toxicology. All biomarkers are also measured in animal models, often aiming to assist the design and interpretation of clinical studies, so it is important to understand the validity of these measurements in preclinical systems. Table 4 includes a representative selection of such MR biomarkers, with simple assessments of how instrumentation-derived irreproducibility in *R*_1_ might propagate. For example, the measured between-centre uncertainty in precontrast *R*_1_ translates to at most 10% between-centre uncertainty in the biomarkers derived from DCE-MRI (Table 4). This error is smaller than the typical day-to-day repeatability error, and in itself would have little effect on the interpretation of change in parameters such as *K*^trans^, because treatment effects are typically much >10% [59].

A realistic assessment of propagation of errors is complex and beyond the scope of this work. In particular, in compartmental models, reproducibility errors and repeatability errors are not completely independent. We omitted from consideration terms which primarily affect repeatability, such as error cancellation with post-contrast *R*_1_, additional *R*_1_ errors that arise in the presence of contrast agent (*e.g.* signal saturation, limited water exchange), and *in vivo* effects (*e.g.* inflow, breathing motion, bolus dispersion, partial volume). Nevertheless, Table 4 provides comparative order-of magnitude assessments to highlight cases in which the variance seen in our study might be important. With this caveat, in myocardial fibrosis, in normal-appearing multiple sclerosis white mater, and in oxygen-enhanced MRI, *R*_1_-based MR biomarkers would be quite sensitive even to such small errors in *R*_1_ unless additional acquisition and analysis methods are designed to reduce the impact of error propagation. An example of this is the use of dynamic time series in OE-MRI that determine ∆*R*_1_(t) by referencing the time-varying signal to a baseline *R*_1_ measurement, thereby reducing the degrees of freedom in the measurement and subsequent error propagation [60]. Similar approaches have been common in DCE-MRI for many years.

### 4.3 Study limitations

(1) This study was performed using only one vendor's equipment, Bruker Avance I, II or III systems running Paravision 5 or 6, representing a typical range of equipment for preclinical MR biomarker research at the time when the study was performed (2017–18). The findings may not be translatable to other vendors' equipment.
(2) Only one pulse sequence (saturation recovery with RARE readout) was employed. This was chosen [61] in a compromise between accuracy and speed. However the assumption of zero longitudinal magnetisation at the mid-point of the eighth echo may be invalid if B_1_ is imperfect, and the findings may not be translatable to other sequences with different B_1_ sensitivity.
(3) The accuracy of our data was not verified using an external standard, such as spectroscopic inversion-recovery.
(4) A common problem for MR phantoms is temperature dependence. In addition to ambient room temperature, heat is imparted to the phantom from the shims during the working day, from the pulsed gradients, and directly from radiofrequency power deposited by the pulse. Data at 1.5 T [4] and 2.35 T [15] show *R*_1_ temperature dependencies in the range −1.3%/°C to +0.7%/°C; data at 0. 08 T [16] show an *r*_1_ temperature dependence of 0.006 s^−1^ mM^−1^/°C. Although temperatures were measured in this study, no direct measurement was made of the agarose temperature itself during MR data acquisition, and exploratory analyses did not reveal temperature as a confound.
(5) In order to address the question of reproducibility in normal academic practice, our study modelled “institution-led” standardisation. No site training was performed, no quality control was imposed, nor were sites permitted to repeat their measurements to eliminate apparent outliers. We did not verify that all scanners were performing optimally, and indeed SNR estimated from the DNE runs did not show the anticipated variation with B_0_ or coil design. RoIs and *T*_1_ calculations were performed locally. Possibly, “centrally-led” standardisation rigorously imposed by a core lab might further improve reproducibility.
(6) No phantom study can fully model the *in vivo* measurement. Nevertheless, a well-designed phantom study sets a lower limit on the error to be expected from measurements in living animals.

### 4.4 Conclusions

Using nickel agarose phantoms in typical preclinical MR systems, *R*_1_ exhibited adequate reproducibility for most purposes. Reproducibility (and repeatability) of <0.06 s^−1^ and < 2.4% was readily achieved. These small technical (instrumentation-derived) errors in *R*_1_ measurement mostly do not contribute biologically significant errors into *R*_1_-based MR biomarkers. However, in a small number of very demanding applications, such as myocardial fibrosis, white mater, or oxygen-enhanced MRI, the accuracy of *R*_1_-based MR biomarkers would be quite sensitive even to such small errors in *R*_1_, therefore in these cases further work may be needed to adequately standardise *R*_1_ data acquisition and analysis.

## Conflicts of interest

CG, GS and SZ are employees of Bayer AG, a for-profit company providing MR contrast agents. PDH is an employee at Antaros Medical, a for-profit company providing MR biomarker services. SK and KS are employees of Bruker BioSpin MRI GmbH, a for-profit company which is the manufacturer of the MR systems used in the study. JCW receives compensation from Bioxydyn Ltd., a for-profit company providing MR biomarker services.

## Appendix A Supplementary data

Supplementary material Image 1
