# Supplementary material for: Repeatability and reproducibility of longitudinal relaxation rate in 12 small-animal MRI systems
Source: Magn Reson Imaging. 2019 Jun;59:121–9. doi: 10.1016/j.mri.2019.03.008 (PMC6477178; doi:10.1016/j.mri.2019.03.008)

Supplementary Material

S1. Author contributions

Name	The author asserts s/he made substantial contributions to the conception or design of the work; or the acquisition, analysis, or interpretation of data for the work	The author asserts s/he contributed to drafting the work or revising it critically for important intellectual content	The author gives final approval of this version to be published	The author agrees to be accountable for all aspects of the work in ensuring that questions related to the accuracy or integrity of any part of the work are appropriately investigated and resolved
Campbell, Simon	Acquiring data	yes	yes	yes
Gottschalk, Michael	Acquiring data	yes	yes	yes
Green, Claudia	Acquiring data	yes	yes	yes
Haase, Michael	Acquiring data	yes	yes	yes
Hines, Catherine DG	Optimising protocol<br>Acquiring data	yes	yes	yes
Hockings, Paul	Optimising protocol<br>Acquiring data	yes	yes	yes
Hoffmann, Katja	Acquiring data	yes	yes	yes
Juretschke, Hans-Paul	Devising protocol	yes	yes	yes
Köhler, Sascha	Devising protocol	yes	yes	yes
Laitinen, Iina	Optimising protocol<br>Acquiring data	yes	yes	yes
Lloyd, William	Acquiring data	yes	yes	yes
Luo, Yanping	Acquiring data	yes	yes	yes
Mahmutovic Persson, Irma	Acquiring data	yes	yes	yes
O'Connor, James PB	Interpreting data	yes	yes	yes
Olsson, Lars E	Devising phantom	yes	yes	yes
Pindoria, Kashmira	Acquiring data	yes	yes	yes
Schneider, Jurgen E.	Acquiring data	yes	yes	yes
Schütz, Gunnar	Devising project<br>Devising phantom<br>Acquiring data	yes	yes	yes
Sourbron, Steven	Simulations	yes	yes	yes
Steinmann, Denise	Acquiring data	yes	yes	yes
Strobel, Klaus	Acquiring data	yes	yes	yes
Tadimalla, Sirisha	Simulations	yes	yes	yes
Teh, Irvin	Acquiring data	yes	yes	yes
Veltien, Andor	Acquiring data	yes	yes	yes
Waterton, John C	Devising project<br>Devising protocol<br>Acquiring data<br>Analysing data<br>Interpreting data	yes	yes	yes
Ziemian, Sabina	Devising phantom<br>Acquiring data	yes	yes	yes
Zhang, Xiaomeng	Acquiring data	yes	yes	yes

S2.1. Raw data

The columns represent: centre; B_0; Phantom [Ni^{2+}]; whether first or second day; R_1 for RoI at isocentre; R_1 for RoI displaced in X; R_1 for RoI displaced in Z; SD for R_1 for RoI at isocentre; SD for R_1 for RoI displaced in X; SD for R_1 for RoI displaced in Z; temperature °C.

```
centre,B0,mM,day,O,X,Z,eO,eX,eZ,T
A,7.0,0.50,1,0.820,0.840,0.893,0.028,0.039,0.067,20.78
A,7.0,0.50,2,0.781,0.787,0.794,0.006,0.008,0.009,20.70
A,7.0,1.04,1,1.267,1.274,1.258,0.015,0.017,0.024,19.70
A,7.0,1.04,2,1.302,1.297,1.332,0.012,0.013,0.015,20.80
A,7.0,2.02,1,2.079,2.110,2.066,0.046,0.037,0.077,21.03
A,7.0,2.02,2,2.101,2.128,2.141,0.024,0.043,0.036,20.70
A,7.0,4.08,1,3.984,3.817,3.861,0.129,0.152,0.136,21.05
A,7.0,4.08,2,3.831,3.774,3.891,0.033,0.038,0.050,20.74
A,7.0,8.05,1,6.667,7.143,7.194,0.782,0.653,0.274,20.50
A,7.0,8.05,2,7.143,7.143,7.194,0.104,0.175,0.093,20.80
B,3.0,0.50,1,0.725,0.730,0.730,0.013,0.014,0.014,20.05
B,3.0,0.50,2,0.794,0.794,0.813,0.013,0.014,0.014,15.00
B,3.0,1.04,1,1.068,1.086,1.100,0.012,0.011,0.012,19.55
B,3.0,1.04,2,1.120,1.115,1.149,0.004,0.008,0.005,16.75
B,3.0,2.02,1,1.764,1.761,1.812,0.005,0.005,0.005,19.80
B,3.0,2.02,2,1.776,1.754,1.805,0.004,0.006,0.004,17.75
B,3.0,4.08,1,3.115,3.115,3.165,0.002,0.002,0.002,19.00
B,3.0,4.08,2,3.125,3.115,3.154,0.004,0.001,0.002,18.15
B,3.0,8.05,1,5.747,5.747,5.780,0.001,0.001,0.001,18.60
B,3.0,8.05,2,5.814,5.747,5.814,0.002,0.001,0.002,18.35
C,7.0,0.50,1,0.788,0.807,0.806,0.006,0.005,0.011,20.80
C,7.0,0.50,2,0.803,0.816,0.824,0.006,0.008,0.007,21.45
C,7.0,1.04,1,1.266,1.292,1.288,0.010,0.010,0.011,20.60
C,7.0,1.04,2,1.255,1.255,1.266,0.008,0.011,0.008,21.55
C,7.0,2.02,1,2.127,2.131,2.160,0.014,0.010,0.012,21.00
C,7.0,2.02,2,2.131,2.104,2.175,0.019,0.017,0.022,21.60
C,7.0,4.08,1,3.838,3.936,3.841,0.030,0.012,0.028,21.40
C,7.0,4.08,2,3.827,3.882,3.844,0.021,0.025,0.011,21.40
C,7.0,8.05,1,7.196,7.227,7.144,0.046,0.040,0.045,21.70
C,7.0,8.05,2,7.248,7.357,7.193,0.043,0.070,0.040,21.25
D,4.7,0.50,1,0.794,0.800,0.813,0.017,0.020,0.019,18.25
D,4.7,0.50,2,0.775,0.794,0.794,0.016,0.024,0.019,19.35
D,4.7,1.04,1,1.174,1.185,1.206,0.029,0.024,0.028,18.90
D,4.7,1.04,2,1.181,1.189,1.206,0.028,0.031,0.031,19.70
D,4.7,2.02,1,1.953,1.953,2.000,0.044,0.045,0.041,19.40
D,4.7,2.02,2,1.931,1.942,1.976,0.044,0.043,0.041,19.65
D,4.7,4.08,1,3.472,3.472,3.521,0.075,0.084,0.077,19.45
D,4.7,4.08,2,3.484,3.484,3.546,0.075,0.098,0.079,19.85
D,4.7,8.05,1,6.410,6.410,6.494,0.177,0.196,0.175,19.35
D,4.7,8.05,2,6.494,6.494,6.536,0.191,0.152,0.175,19.65
E,7.0,0.50,1,0.800,0.826,0.826,0.013,0.021,0.016,17.80
E,7.0,0.50,2,0.794,0.813,0.813,0.015,0.015,0.020,18.50
E,7.0,1.04,1,1.255,1.276,1.287,0.028,0.052,0.034,18.35
E,7.0,1.04,2,1.261,1.290,1.300,0.021,0.038,0.029,19.85
E,7.0,2.02,1,2.096,2.183,2.183,0.042,0.038,0.053,18.35
E,7.0,2.02,2,2.083,2.160,2.146,0.032,0.041,0.044,20.55
E,7.0,4.08,1,3.802,3.984,3.891,0.070,0.083,0.073,18.65
E,7.0,4.08,2,3.788,3.906,3.876,0.065,0.085,0.074,20.30
E,7.0,8.05,1,7.143,7.519,7.353,0.155,0.205,0.234,19.25
E,7.0,8.05,2,6.993,7.463,7.299,0.185,0.217,0.214,19.75
F,7.0,0.50,1,0.810,0.820,0.813,0.008,0.007,0.009,20.50
F,7.0,0.50,2,0.806,0.817,0.820,0.006,0.005,0.009,19.20
F,7.0,1.04,1,1.272,1.294,1.285,0.011,0.012,0.015,18.85
F,7.0,1.04,2,1.245,1.267,1.284,0.017,0.014,0.018,19.50
F,7.0,2.02,1,2.119,2.146,2.155,0.009,0.009,0.014,19.45
F,7.0,2.02,2,2.151,2.165,2.155,0.018,0.019,0.019,19.00
F,7.0,4.08,1,3.891,3.953,3.937,0.030,0.031,0.062,18.95
F,7.0,4.08,2,3.906,3.937,3.937,0.046,0.078,0.031,18.85
F,7.0,8.05,1,7.299,7.299,7.353,0.053,0.053,0.054,19.10
```

```
F,7.0,8.05,2,7.407,7.407,7.353,0.165,0.055,0.108,18.65
G1,7.0,0.50,1,0.817,0.812,0.796,0.007,0.006,0.005,18.25
G1,7.0,0.50,2,0.820,0.833,0.817,0.002,0.002,0.002,18.20
G1,7.0,1.04,1,1.260,1.290,1.284,0.015,0.009,0.010,18.35
G1,7.0,1.04,2,1.250,1.269,1.231,0.006,0.005,0.008,18.40
G1,7.0,2.02,1,2.114,2.164,2.117,0.048,0.054,0.053,18.40
G1,7.0,2.02,2,2.112,2.182,2.096,0.053,0.053,0.051,18.40
G1,7.0,4.08,1,3.909,3.956,3.881,0.097,0.109,0.099,18.50
G1,7.0,4.08,2,3.905,3.904,3.902,0.100,0.114,0.101,18.35
G1,7.0,8.05,1,7.193,7.456,7.345,0.234,0.249,0.261,18.45
G1,7.0,8.05,2,7.197,7.196,7.144,0.227,0.209,0.216,18.15
G2,4.7,0.50,1,0.748,0.756,0.767,0.037,0.039,0.037,18.45
G2,4.7,0.50,2,0.769,0.773,0.782,0.035,0.037,0.035,18.15
G2,4.7,1.04,1,1.143,1.158,1.171,0.048,0.049,0.053,18.25
G2,4.7,1.04,2,1.132,1.152,1.170,0.040,0.051,0.049,18.30
G2,4.7,2.02,1,1.883,1.883,1.943,0.098,0.088,0.083,18.15
G2,4.7,2.02,2,1.873,1.884,1.936,0.073,0.076,0.084,18.15
G2,4.7,4.08,1,3.358,3.479,3.476,0.150,0.165,0.159,18.20
G2,4.7,4.08,2,3.439,3.464,3.490,0.153,0.159,0.149,18.20
G2,4.7,8.05,1,6.370,6.336,6.428,0.319,0.343,0.346,18.10
G2,4.7,8.05,2,6.437,6.412,6.484,0.335,0.356,0.341,18.35
H,4.7,0.50,1,0.761,0.769,0.757,0.007,0.007,0.008,20.75
H,4.7,0.50,2,0.749,0.748,0.733,0.005,0.005,0.008,21.55
H,4.7,1.04,1,1.155,1.144,1.163,0.010,0.010,0.013,21.25
H,4.7,1.04,2,1.160,1.126,1.148,0.016,0.013,0.016,21.30
H,4.7,2.02,1,1.942,1.912,1.936,0.008,0.008,0.013,21.05
H,4.7,2.02,2,1.922,1.910,1.954,0.017,0.017,0.017,20.95
H,4.7,4.08,1,3.461,3.453,3.461,0.027,0.027,0.055,20.90
H,4.7,4.08,2,3.517,3.411,3.514,0.041,0.067,0.028,20.90
H,4.7,8.05,1,6.464,6.443,6.592,0.047,0.047,0.048,20.85
H,4.7,8.05,2,6.566,6.373,6.553,0.146,0.047,0.096,20.90
J,4.7,0.50,1,0.789,0.842,0.795,0.010,0.014,0.011,NaN
J,4.7,0.50,2,0.798,0.798,0.808,0.006,0.007,0.008,NaN
J,4.7,1.04,1,1.183,1.276,1.187,0.007,0.024,0.009,NaN
J,4.7,1.04,2,1.187,1.196,1.213,0.010,0.008,0.013,NaN
J,4.7,2.02,1,1.956,1.966,1.985,0.014,0.057,0.040,NaN
J,4.7,2.02,2,1.934,1.941,1.954,0.011,0.016,0.015,NaN
J,4.7,4.08,1,3.419,3.572,3.402,0.013,0.043,0.038,NaN
J,4.7,4.08,2,3.512,3.454,3.564,0.017,0.024,0.022,NaN
J,4.7,8.05,1,6.237,6.665,6.187,0.066,0.070,0.084,NaN
J,4.7,8.05,2,6.433,6.370,6.478,0.073,0.038,0.046,NaN
K,9.4,0.50,1,0.862,0.877,0.862,0.017,0.015,0.018,18.05
K,9.4,0.50,2,0.885,0.847,0.862,0.014,0.013,0.013,18.85
K,9.4,1.04,1,1.401,1.381,1.397,0.024,0.025,0.021,18.40
K,9.4,1.04,2,1.387,1.379,1.366,0.023,0.021,0.022,18.50
K,9.4,2.02,1,2.336,2.326,2.342,0.033,0.065,0.038,18.05
K,9.4,2.02,2,2.336,2.304,2.331,0.038,0.036,0.037,18.80
K,9.4,4.08,1,4.202,4.237,4.167,0.071,0.108,0.069,18.05
K,9.4,4.08,2,4.202,4.149,4.202,0.064,0.074,0.076,18.85
K,9.4,8.05,1,7.874,7.634,7.813,0.124,0.175,0.183,18.05
K,9.4,8.05,2,7.874,7.752,7.813,0.155,0.162,0.177,18.85
L,11.7,0.50,1,0.870,0.885,0.885,0.011,0.013,0.024,18.15
L,11.7,0.50,2,0.916,0.910,0.919,0.011,0.012,0.018,18.15
L,11.7,1.04,1,1.266,1.284,1.252,0.034,0.031,0.019,18.60
L,11.7,1.04,2,1.462,1.490,1.479,0.024,0.023,0.030,18.45
L,11.7,2.02,1,2.353,2.358,2.347,0.300,0.322,0.326,18.65
L,11.7,2.02,2,2.564,2.513,2.475,0.048,0.053,0.053,18.05
L,11.7,4.08,1,4.274,4.274,4.329,0.069,0.071,0.071,17.30
L,11.7,4.08,2,4.348,4.292,4.367,0.079,0.074,0.071,18.20
L,11.7,8.05,1,7.937,8.000,8.065,0.157,0.147,0.150,18.65
L,11.7,8.05,2,7.937,8.000,8.065,0.151,0.147,0.150,17.75
```

S2.2. Raw data DNE

The columns represent the mean ÷ SD of the 38 measurements from RoI {0,0,0} made dynamically over 5 minutes in each of the centres

day	mM	A	B	C	D	E	F	G1	G2	H	J	K	L
1	0.5	231	143	328	231	183	234	363	139	73	NaN	269	40
1	1	461	190	295	231	253	372	417	199	89	NaN	207	60
1	2	484	291	306	277	212	350	166	220	104	NaN	313	51
1	4	227	299	424	319	194	415	175	237	95	NaN	182	53
1	8	152	300	357	307	224	309	187	281	98	NaN	231	49
2	0.5	112	230	242	170	157	259	281	154	66	NaN	282	32
2	1	92	185	322	221	234	281	207	123	109	NaN	319	41
2	2	435	206	391	287	238	348	284	183	125	NaN	303	66
2	4	603	209	335	300	114	403	273	222	127	NaN	530	42
2	8	127	204	415	286	204	359	401	215	122	NaN	244	72

S3. Phantoms

Preparation and validation of the phantom material

Materials: nickel chloride hexahydrate (purity 99.99%), agarose molecular biology reagent, sodium azide (purity 99.5%), paraffin wax (mp 53-57 °C, ASTM D 87) were all purchased from Sigma-Aldrich (Sigma-Aldrich Chemie GmbH, Steinheim). Water was purified by a Milli-Q Gradient machine (Merck KGaA, Darmstadt, Germany) equipped with a 0.16 µm filter.

Phantoms with five nickel chloride concentrations of 0.5, 1, 2, 4 and 8 mM, 2 wt% agarose and 0.05 wt% sodium azide were prepared in glass vials with dimensions of 30 x 55 mm. The diameter of the vial was chosen so that the vial can be readily imaged in a standard pre-clinical MRI scanner.

Each phantom nickel concentration preparation was executed separately in a total volume of 0.5 L. 10 g of agarose and 0.25 g of sodium azide were added to nickel chloride hexahydrate (0.0594, 0.1188, 0.2377, 0.4753 and 0.9506 g for the preparation of 0.5, 1, 2, 4 and 8 mM, respectively) dissolved in 400 mL of MilliQ water in a 0.5 L glass round bottom flask. The mixture was subjected to a microwave at 600 W up to 7 min to dissolve agarose but not allowing for a vigorous boiling. Once dissolved, the mixture was transferred to a 0.5 L Erlenmeyer flask with ground glass stopper. The total volume of phantom material was adjusted to 0.5 L by an addition of Millipore water (at approx. 80 °C). Then the mixture was shaken and placed in an ultrasonic bath set at 80 °C for 10 min. The final solution was poured into 20 vials to the height of 4 cm. The vials were closed with black gummi lids and positioned in a tray in the ultrasonic bath for 3 min at 80 °C. The phantom material was left to solidify. After 2 h, a melted paraffin wax was poured on top of an agarose gel forming a thin layer. The vials were covered with the gummi lids on top of a hot paraffin layer.

Phantom No	Theor Ni Conc (mM)	Vol (l)	Ni mass (g)	2 wt% Agarose (g)	0,05 wt% sodium azide (g)
1	0,50	0,5	0,0594	10	0,25
2	1,00	0,5	0,1188	10	0,25
3	2,00	0,5	0,2377	10	0,25
4	4,00	0,5	0,4753	10	0,25
5	8,00	0,5	0,9506	10	0,25

Nickel concentration was analysed by ICP-OES . One vial for each set of phantoms was randomly picked up and nickel concentration was measured at 3 different heights for each vial.

Calibration standards: 5 ppm Ni + 2 ppm Y in 5% HNO_3.

All samples were treated as tissue samples. 100 mg gel + Y (internal standard, sufficient amount for the final volume to be 2 ppm) and 65% HNO3 + 30% H_2O_2 were added according to large vessel degradation procedure KM A 10. Final volume was diluted so that ~ 2-10 ppm Ni in ~ 5% HNO_3 was obtained.

Phantom No	Measured Ni Conc. (mM) by ICP-OES
1	0,50 ± 0,01
2	1,04 ± 0,03
3	2,02 ± 0,03
4	4,08 ± 0,04
5	8,05 ± 0,12

Supplementary Figure 3.1

R_1 phantoms

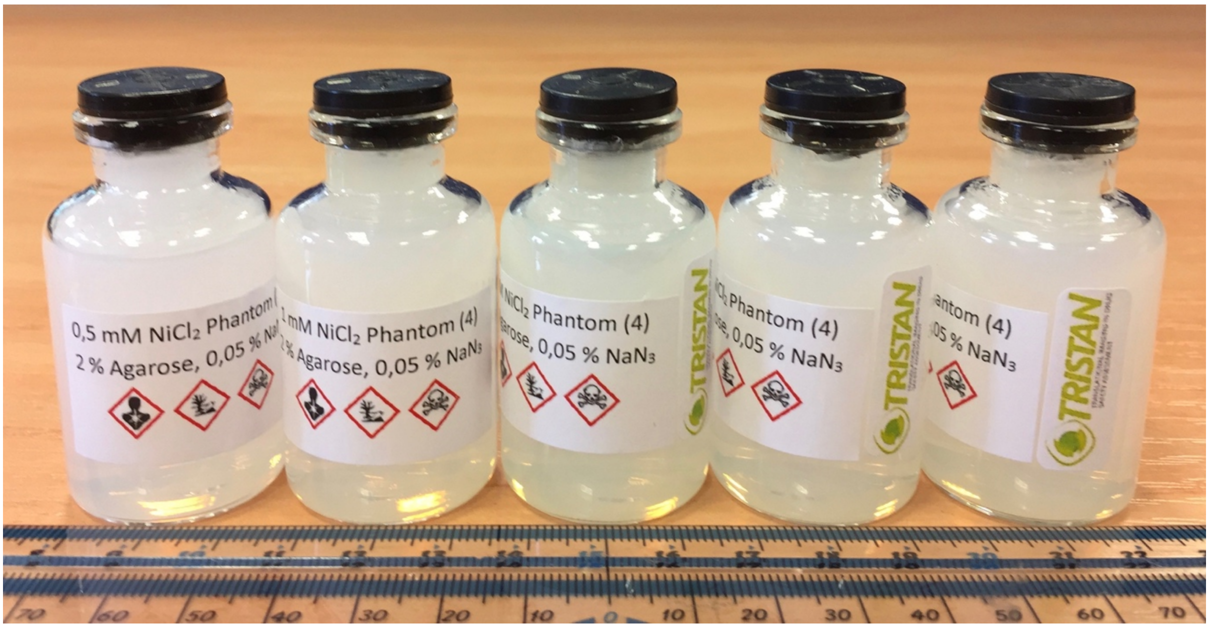

Supplementary Figure 3.2

R_1 phantom *in situ* in cucumber

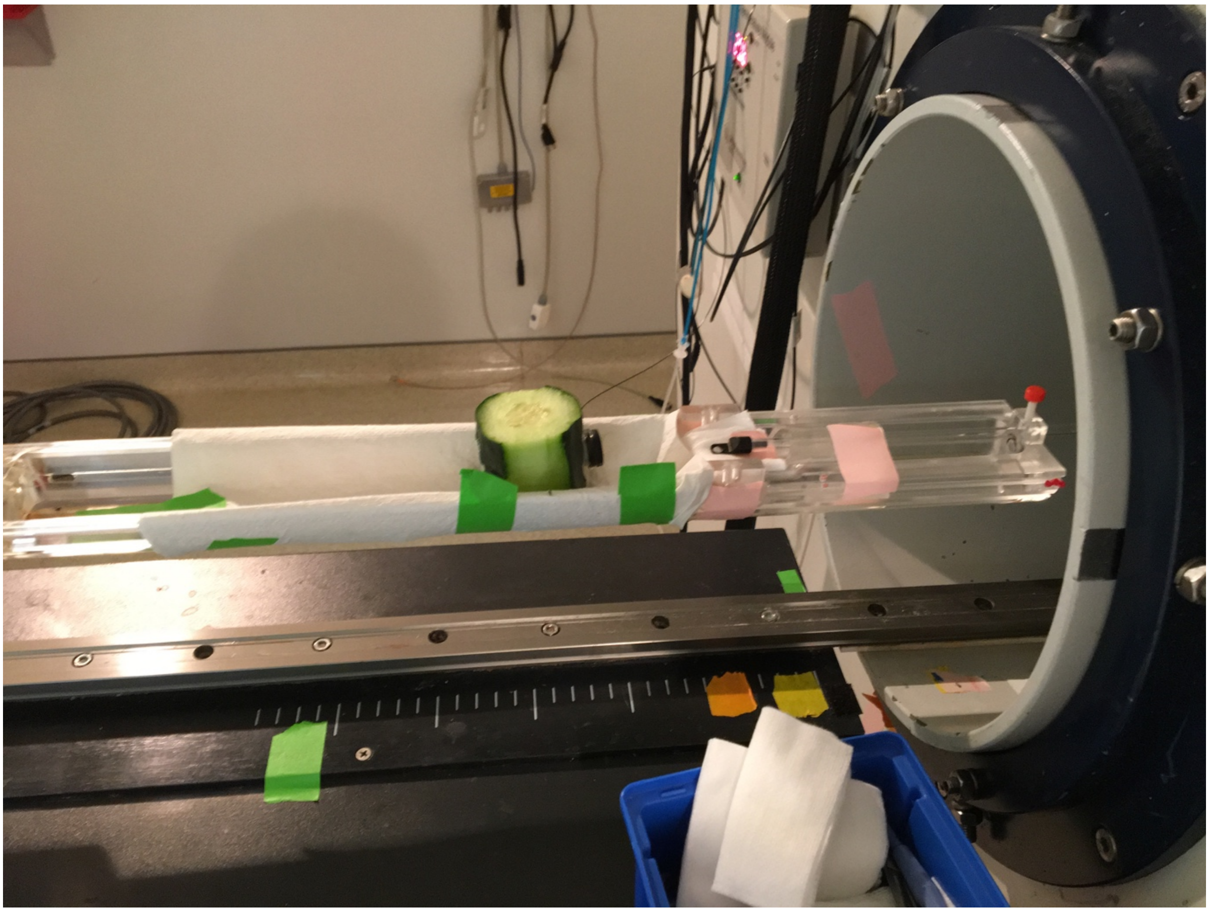

Supplementary Figure 3.3

R_1 map of phantom *in situ* in cucumber

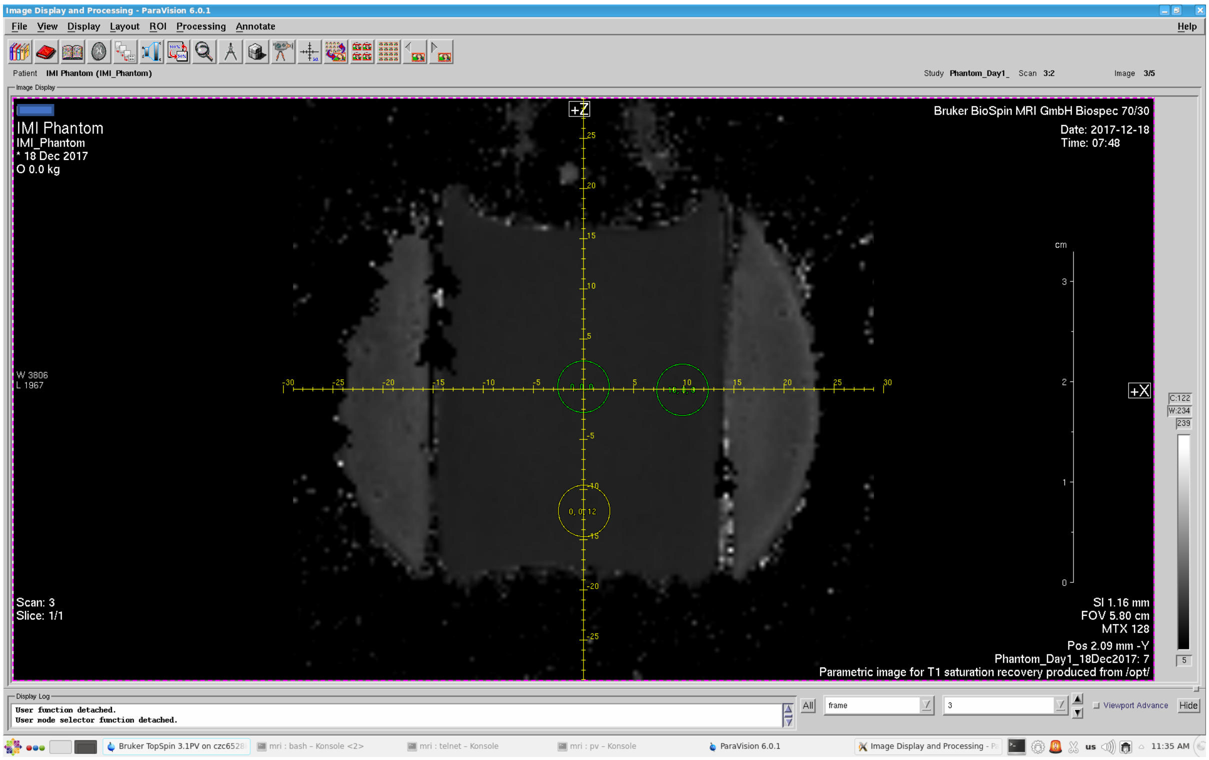

S4. Error propagation in compartmental models:

Error propagation from pre-contrast R_1 to DCEMRI biomarkers was simulated for four preclinical case-studies, as shown in Table 4. Representative 'true' values of kinetic parameters, pre-contrast R_1 values ($R_{1,0}$), and appropriate tracer kinetic models were chosen from literature to estimate contrast agent concentration uptake in each tissue type. A population-based input function, given by Model B in [1], was assumed. A fixed error in $R_{1,0}$ (calculated as $2\times SD = 0.0624\ s^{-1}$) was propagated to the dynamic MR signal assuming a spoiled gradient acquisition. The signal was then converted to a concentration profile, and tracer kinetic model fitting was used to estimate kinetic parameters. Absolute errors in kinetic parameters are given in Table 4.

Note that the $R_{1,0}$ error propagation simulated in this paper does not consider the effects of errors or error compensation in other data. In particular we assume that the dynamic $R_{1,t}$ measurements are error-free.

References

1. McGrath, D. M.; Bradley, D. P.; Tessier, J. L.; Lacey, T.; Taylor, C. J.; Parker, G. J. M. Comparison of Model-Based Arterial Input Functions for Dynamic Contrast-Enhanced MRI in Tumor Bearing Rats. *Magn. Reson. Med.* **2009**, *61* (5), 1173–1184.
2. Larsson, C.; Kleppestø, M.; Grothe, I.; Vardal, J.; Bjørnerud, A. T1 in High-Grade Glioma and the Influence of Different Measurement Strategies on Parameter Estimations in DCE-MRI. *J. Magn. Reson. Imaging* **2015**, *42* (1), 97–104.
3. Merali, Z.; Huang, K.; Mikulis, D.; Silver, F.; Kassner, A. Evolution of Blood-Brain-Barrier Permeability after Acute Ischemic Stroke. *PLoS One* **2017**, *12* (2), e0171558.
4. Jakob, P. M.; Hillenbrand, C. M.; Wang, T.; Schultz, G.; Hahn, D.; Haase, A. Rapid Quantitative lung1H T1 Mapping. *J. Magn. Reson. Imaging* **2001**, *14* (6), 795–799.
5. Chow, A. M.; Gao, D. S.; Fan, S. J.; Qiao, Z.; Lee, F. Y.; Yang, J.; Man, K.; Wu, E. X. Measurement of Liver T1 and T2 Relaxation Times in an Experimental Mouse Model of Liver Fibrosis. *J. Magn. Reson. Imaging* **2012**, *36* (1), 152–158.
6. Chwang, W. B.; Jain, R.; Bagher-Ebadian, H.; Nejad-Davarani, S. P.; Iskander, A. S. M.; VanSlooten, A.; Schultz, L.; Arbab, A. S.; Ewing, J. R. Measurement of Rat Brain Tumor Kinetics Using an Intravascular MR Contrast Agent and DCE-MRI Nested Model Selection. *J. Magn. Reson. Imaging* **2014**, *40* (5), 1223–1229.
7. Abo-Ramadan, U.; Durukan, A.; Pitkonen, M.; Marinkovic, I.; Tatlisumak, E.; Pedrono, E.; Soinne, L.; Strbian, D.; Tatlisumak, T. Post-Ischemic Leakiness of the Blood–brain Barrier: A Quantitative and Systematic Assessment by Patlak Plots. *Exp. Neurol.* **2009**, *219* (1), 328–333.
8. Mistry, N. N.; Qi, Y.; Hedlund, L. W.; Johnson, G. A. Ventilation/perfusion Imaging in a Rat Model of Airway Obstruction. *Magn. Reson. Med.* **2010**, *63* (3), 728–735.
9. Ulloa, J. L.; Stahl, S.; Yates, J.; Woodhouse, N.; Kenna, J. G.; Jones, H. B.; Waterton, J. C.; Hockings, P. D. Assessment of Gadoxetate DCE-MRI as a Biomarker of Hepatobiliary Transporter Inhibition. *NMR Biomed.* **2013**, *26* (10), 1258–1270.
10. Hockings, P.; Karageorgis, A.; Lenhard, S.; Yerby, B.; Forsgren, M.; Liachenko, S.; Johansson, E.; Peterson, R.; Yang, X.; Williams, D.; et al. A Multicenter in Vivo Study to Evaluate Gadoxetate DCE-MRI as a Preclinical Biomarker of Liver Function. *ISMRM Annual Meeting* **2017**, Abstract No. 4862.

Supplementary Table 4.1: values used to populate Table 4:

Case-study	Parameters		Absolute error $\lvert X_{R1,0error} - X_{true} \rvert$ [a]	Relative error (%) $100\frac{\lvert X_{R1,0error} - X_{true} \rvert}{X_{true}}$
Glioma	$R_{1,0}$ (s^{-1}) [2]	0.67	0.0624	9.3
	v_p	0.016	0.0016	9.7
	v_e	0.236	0.0239	10.1
	K_{trans} (min^{-1})	0.046	0.0039	8.5
Transient ischemia	$R_{1,0}$ (s^{-1}) [3]	1.25	0.0624	5.0
	v_p	0.015	0.0008	5.2
	K_i ($ml.g^{-1}. s^{-1}$)	0.0035	0.0002	5.3
Normal lung	$R_{1,0}$ (s^{-1}) [4]	0.77	0.0624	8.1
	v_p	0.419	0.0437	10.4
	F_p (min^{-1})	0.385	0.0299	7.8
Normal hepatocyte transporters	$R_{1,0}$ (s^{-1}) [5]	1.05	0.0624	5.9
	v_e	0.23	0.0163	7.1
	k_1 ($mM.s^{-1}$)	0.0294	0.0013	4.5
	k_2 (s^{-1})	0.0049	0.0001	1.7

[a] $X_{R1,0error}$ and X_{true} represent the biomarker value with and without an error in $R_{1,0}$

Supplementary Table 4.2: tracer kinetic model and DCEMRI parameters used in the simulations

Case-study	Model parameters	DCEMRI parameters
Glioma [6]	Extended Tofts:<br>$$C(t) = v_p c_a(t) + K_{trans} e^{\left(-t\frac{K_{trans}}{v_e}\right)} * c_a(t)$$<br>$v_p = 0.67$; $v_e = 0.016$; $K_{trans} = 0.046$	TR = 5.65 ms<br>$\alpha = 30°$<br>temporal resolution = 11.57 s<br>DCEMRI scan time = 15 min<br>r_1 (gadopentetate) = 3.9 $s^{-1}.mM^{-1}$
Transient Ischemia [7]	Patlak:<br>$$C(t) = v_p c_a(t) + K_i \int_0^t c_a(t)dt$$<br>$v_p = 0.015$; $K_i = 0.0035$	TR = 5.65 ms<br>$\alpha = 30°$<br>temporal resolution = 60 s<br>DCEMRI scan time = 30 min<br>r_1 (gadopentetate) = 3.8 $s^{-1}.mM^{-1}$
Normal lung [8]	Model-free deconvolution:<br>$$C(t) = c_a(t) * h(t)$$<br>$$v_p = \int_0^t h(t)dt; \quad F_p = \max(h(t))$$<br>$v_p = 0.419$; $F_p = 0.385$	TR = 4 ms<br>$\alpha = 40°$<br>temporal resolution = 0.4 s<br>DCEMRI scan time = 45 min<br>r_1 (gadopentetate) = 3.8 $s^{-1}.mM^{-1}$
Normal hepatocyte transporters [9][10]	2-compartment uptake and efflux:<br>$$C(t) = v_e c_a(t) + k_1(1 - v_e)e^{(-tk_2)} * c_a(t)$$<br>$v_e = 0.23$; $k_1 = 0.0294$; $k_2 = 0.0049$	TR = 7.1 ms<br>$\alpha = 30°$<br>temporal resolution = 60 s<br>DCEMRI scan time = 60 min<br>r_1 (gadoxetate) = 5.9 $s^{-1}.mM^{-1}$

S5. Comparisons of R_1 fits in “R” and ParaVision

Comparison between 2-parameter R_1 analysis performed locally in ParaVision (R_1^{PV}) and R_1 analyses performed centrally in “R” (R_1^{R}). Each panel shows a plot of $\log_{10} R_1^{\mathrm{PV}} - \log_{10} R_1^{\mathrm{R}}$ against $\left(\log_{10} R_1^{\mathrm{PV}} + \log_{10} R_1^{\mathrm{R}}\right)/2$. Error bars are derived from the Standard Error in R_1 from the `nls()` fit in “R”.

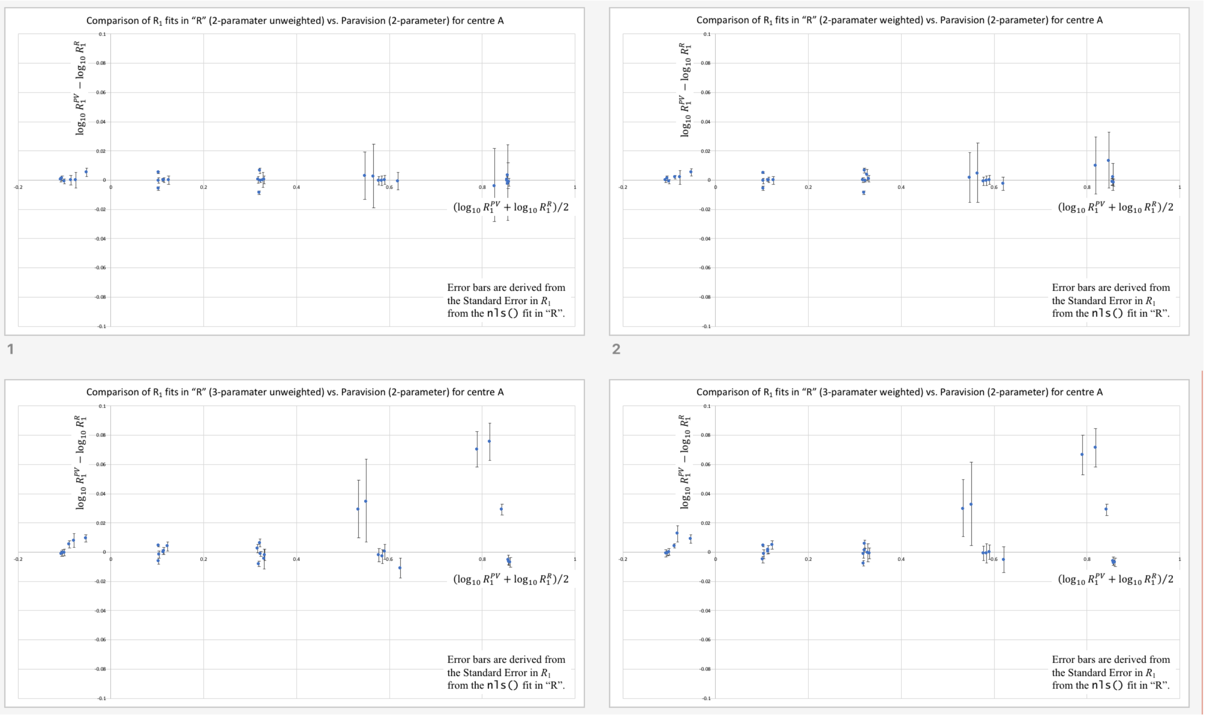

Supplement: Supplementary file 1 — Supplementary material [file mmc1.pdf]
